# Nuclear entry and egress of parvoviruses

**DOI:** 10.1111/mmi.14974

**Published:** 2022-08-24

**Authors:** Salla Mattola, Vesa Aho, Luisa F. Bustamante‐Jaramillo, Edoardo Pizzioli, Michael Kann, Maija Vihinen‐Ranta

**Affiliations:** ^1^ Department of Biological and Environmental Science University of Jyvaskyla Jyvaskyla Finland; ^2^ Department of Infectious Diseases, Institute of Biomedicine University of Gothenburg Gothenburg Sweden; ^3^ Sahlgrenska Academy Gothenburg Sweden; ^4^ Department of Clinical Microbiology Region Västra Götaland, Sahlgrenska University Hospital Gothenburg Sweden

**Keywords:** import and export, nuclear envelope, nuclear pore complexes, nucleus, parvoviruses

## Abstract

Parvoviruses are small non‐enveloped single‐stranded DNA viruses, which depend on host cell nuclear transcriptional and replication machinery. After endosomal exposure of nuclear localization sequence and a phospholipase A_2_ domain on the capsid surface, and escape into the cytosol, parvovirus capsids enter the nucleus. Due to the small capsid diameter of 18–26 nm, intact capsids can potentially pass into the nucleus through nuclear pore complexes (NPCs). This might be facilitated by active nuclear import, but capsids may also follow an alternative entry pathway that includes activation of mitotic factors and local transient disruption of the nuclear envelope. The nuclear entry is followed by currently undefined events of viral genome uncoating. After genome release, viral replication compartments are initiated and infection proceeds. Parvoviral genomes replicate during cellular S phase followed by nuclear capsid assembly during virus‐induced S/G2 cell cycle arrest. Nuclear egress of capsids occurs upon nuclear envelope degradation during apoptosis and cell lysis. An alternative pathway for nuclear export has been described using active transport through the NPC mediated by the chromosome region maintenance 1 protein, CRM1, which is enhanced by phosphorylation of the N‐terminal domain of VP2. However, other alternative but not yet uncharacterized nuclear export pathways cannot be excluded.

## Graphical abstract

Parvovirus capsids enter the nucleus by active import or by an alternative entry pathway that includes local transient disruption of the nuclear envelope. Parvoviral genomes replicate during cellular S phase followed by nuclear capsid assembly. The egress of progeny capsids occurs upon nuclear envelope degradation during cell lysis or by using active transport through the nuclear pore complexes.

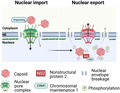

## 1 INTRODUCTION

### 1.1 Parvoviruses

*Parvovirinae* subfamily infects vertebrates including humans. Most viruses of this subfamily, including minute virus of mice (MVM), canine parvovirus (CPV), and rat parvovirus (H‐1PV), are autonomous, but replication of adeno‐associated viruses (AAV) requires the presence of helper viruses such as adenoviruses or herpesviruses (dependoparvoviruses) (Cotmore et al., 2019; Pénzes et al., 2020). While *Parvovirinae* have an important potential in oncolytic therapy, AAVs are a major platform in gene therapy. H‐1PV and MVM are known to induce lysis of transformed cells and to activate anticancer immune responses (Abschuetz et al., 2006; Geletneky et al., 2017; Gil‐Ranedo et al., 2021; Grekova et al., 2012; Hartley et al., 2020; Marchini et al., 2015). The role of CPV in inducing an antitumor immune response in different tumor models has been discussed (Arora et al., 2021). The potential of recombinant AAV vectors in gene therapies has been shown by the approval of two AAV therapeutic applications for the treatment of Leber's congenital amaurosis (Luxturna) and spinal muscular atrophy (Zolgensma) by the US Food and Drug Administration (FDA) (Kuzmin et al., 2021; Large et al., 2021). In fact, the first gene therapy, Glybera medicine, approved in 2012 corrected hereditary lipoprotein lipase deficiency (LPLD). This treatment was stopped in 2018 due to the high cost of c. one million US$ per patient, and only 31 people were treated (Mendell et al., 2021).

Parvoviruses comprise a linear single‐stranded DNA of ~4 to 6 kb and an icosahedral capsid of 18–26 nm in diameter (Cotmore et al., 1983; Kaufmann et al., 2004; Mietzsch et al., 2019; Tsao et al., 1991; Xie et al., 2002). The viral proteome differs between members of parvovirus (Cotmore & Tattersall, 2014). Many of the autonomous parvovirus genome encodes two structural proteins (VP1 and VP2) and two non‐structural proteins (NS1 and NS2) (Cotmore et al., 1983; Cotmore & Tattersall, 2014), whereas AAV encodes at least for three capsid proteins (VP1, VP2, and VP3) and four non‐structural proteins (Rep40, Rep52, Rep68, and Rep78) (Im & Muzyczka, 1990; Xie et al., 2002). Capsid proteins and non‐structural proteins are translated from alternatively spliced mRNAs, following transcription controlled by the early P4 and the late P38 promoter. While the former guides the expression of NS1 and NS2, the latter controls the expression of capsid proteins (Christensen et al., 1995; Cotmore & Tattersall, 1995; Li & Rhode 3rd, 1990). Nonetheless, the family shows different transcriptional strategies and viruses within the type species of each genus express a small number of genus‐specific ancillary proteins (Cotmore & Tattersall, 2014).

Parvoviruses use a variety of cell surface receptors for attachment to their host cells, determining host range and tissue tropism (Govindasamy et al., 2003; Hueffer et al., 2003; Llamas‐Saiz et al., 1996; Michelfelder & Trepel, 2009; Palermo et al., 2006). CPV uses sialic acid and transferrin receptor (Parker et al., 2001; Parrish, 1990), whereas human parvovirus B19V attaches to erythrocyte P antigen (Brown et al., 1993) and its cellular entry is facilitated by low pH‐mediated interaction with globoside (Bieri et al., 2021; Bieri & Ros, 2019). The dependoparvovirus AAV2 recognizes several receptors of target cells including heparan sulfate proteoglycan, αVβ5 integrin, and basic fibroblast growth factor receptor 1 (Qing et al., 1999; Summerford et al., 1999; Summerford & Samulski, 1998). Recently, the AAV receptor (AAVR; KIAA0319L) was identified as an essential receptor for cell internalization and trafficking of different AAVs (Meyer & Chapman, 2022; Pillay et al., 2016). After receptor binding, many parvoviruses enter cells via clathrin‐mediated endocytosis (Bartlett et al., 2000; Cureton et al., 2012; Parker & Parrish, 2000). The low endosomal pH induces conformational changes in parvovirus capsid structure, which leads to exposure of the VP1 N‐terminal unique region (VP1u). VP1u of B19, MVM, and CPV comprises a phospholipase A_2_ (PLA_2_) motif, a nuclear localization sequence (NLS), and three PDZ domains, which are highly conserved. The PLA_2_ domain is required for capsid escape from endocytic vesicles (Farr et al., 2005; Popa‐Wagner et al., 2012; Qu et al., 2008; Suikkanen, Antila, et al., 2003b; Zádori et al., 2001;Ros et al., 2020) presumably by forming holes in the endosomal membrane, while NLS and PDZ domains are implicated in nuclear import of the capsid.

This review focuses on what has been learned in the past years about cytoplasmic trafficking, nuclear entry, and exit of parvovirus capsids.

## 2 NUCLEAR ENTRY OF PARVOVIRUS CAPSIDS

### 2.1 Traveling to the nucleus

Subsequent to endosomal escape, the capsids have to reach the nuclear envelope (NE). Likely, parvoviruses make use of the cellular microtubule network, as depolymerization of microtubules blocks CPV infection (Suikkanen, Aaltonen, et al., 2003a), which is also consistent with their observed velocity toward the nucleus (Mäntylä et al., 2018). These findings are supported by observations on AAV, showing that their perinuclear accumulation is enhanced by dynein‐ and microtubule‐mediated transport (Kelkar et al., 2004, 2006; Xiao & Samulski, 2012). As with CPV, tracking of single AAV particles in the cytoplasm has demonstrated directed motion of viral capsid toward the nucleus, which is a characteristic of dynein‐microtubule mediated transport (Seisenberger et al., 2001).

Direct transport of the released capsids along microtubules is likely but its requirement is not unequivocally proven as microtubule depolymerization does not affect CPV distribution after microinjection (Lyi et al., 2014), which was also observed for cells transduced with recombinant AAV2 vectors (rAAV) (Hirosue et al., 2007). Even less understood is the observation that the intermediate filament protein vimentin enhances infection after endosomal escape as shown for MVM (Fay & Panté, 2013) as intermediate filaments are not polarized and thus hardly contribute to directed cytoplasmic transport. The observation showing that vimentin filaments become disrupted at 14 to 24 h post‐MVM infection, well after nuclear entry should have been completed, indicates an independent phenomenon that is unrelated to early infection events (Nüesch et al., 2005).

Further, some parvoviruses such as MVM and CPV exploit ubiquitin‐proteasome machinery to enhance their nuclear translocation. The presence of a proteosomal inhibitor (MG132) leads to cytoplasmic perinuclear retainment of capsids. However, the viral entry, the natural proteolytic cleavage of VP2 to VP3 and the externalization of the N terminal of VP1 are not affected (Ros & Kempf, 2004). In contrast, ubiquitination of AAV capsids leads to their degradation, and treatment with MG132 increases AAV‐2 and AAV‐5 transduction (Ding et al., 2003; Douar et al., 2001; Yan et al., 2002; Zhong et al., 2008). It remains an open question if the involvement of proteasomes affects cytosolic transport or a subsequent step.

### 2.2 Overview of NPC and nuclear import

Many viruses have adapted to replicate in the host's nuclei, allowing exploitation of cellular machinery like DNA or RNA polymerases. This requires access to the nucleoplasm, which has led to the evolution of specific mechanisms for reaching this compartment. Amongst the best‐described approaches to enter the nucleus are interactions with nuclear pore complexes (NPC) (Fay & Panté, 2015; Guedán et al., 2021).

NPCs are macromolecular structures crossing the NE, allowing passive diffusion only of metabolites and proteins smaller than 30–60 kDa, dependent upon their charge. However, a slow diffusion of larger molecules up to 230 kDa through the NPC has been observed (Popken et al., 2015; Timney et al., 2016; Wang & Brattain, 2007). The NPC is composed by approximately 30 proteins termed nucleoporins (Nups). The shape of the NPC opening is determined by the Y complexes (Nup107‐Nup160 complex) (Stuwe et al., 2015). These are crucial for their interactions with the gel‐like mesh of highly disordered nucleoporins present in the NPC channel, which are characterized by high abundance of short stretches of hydrophobic amino acids comprising phenylalanine (F) and glycine (G) residues. FG‐Nups, which include Nup62, regulate which molecules may traverse the NE and fix cytoplasmic and nuclear fibers extruding from the central part of the NPC (Lyngdoh et al., 2021). Other nucleoporins like Nup153 and Tpr form a basket‐like structure on the nucleoplasmic side, which is necessary for import and export. Many Nups such as Nup153 and Nup62 are also involved in other, non‐transport‐related functions, such as chromosome alignment and binding (Chien et al., 2020; Hashizume et al., 2013). Nup153 and Nup358 have been reported to possess conserved zinc finger domains, which are required for recruitment of coat protein I complex (COPI) coatomers in the early process of nuclear envelope breakdown (NEBD) during mitosis (Liu et al., 2003; Prunuske et al., 2006).

Transport of large proteins or nucleoprotein complexes through the NPC is energy dependent and requires exposure of specific signaling motifs on cargo surface. Classical nuclear localization signals (NLSs) are characterized by short stretches of positively charged amino acids (Arginine and Lysine) exemplified by that of SV40 (PKKKRKV) (Kalderon et al., 1984). Other signals are proline‐tyrosine NLSs, previously termed M9 domains, which comprise highly disordered sequences of 20–30 amino acids interspaced by hydrophobic or basic residues, as, for example, found in hnRNP A1 (Bradley et al., 2007; Görlich, 1997). Not all NLSs are permanently exposed. The so‐called cryptic NLSs become exposed only upon post‐translational modifications or protein–protein interactions (Fagerlund et al., 2002; Gu et al., 2003).

The different signals for nuclear transport through the NPC allow binding of specialized transport receptors, named importins, which are divided into importin α and β (also known as KPNA, KPNB) (Cautain et al., 2015). Of the former, seven members, all involved in nuclear import, are known to serve as adaptor proteins between the nuclear import signal on the cargo and importin β to which it binds via an importin‐binding domain. Depending on the species, between 14 and 20 importin βs have been described. Eleven members of human importins βs facilitate nuclear import. These include transportin (TNPO, also called importin β2), six nuclear export, and three nuclear import and export (Kimura & Imamoto, 2014; Oldrini et al., 2017). Importin β not only binds to cargos via importin α but may also directly interact with cargo‐exposed importin‐binding domains (Lee et al., 2006; Mitrousis et al., 2008). There is, however, growing evidence that nuclear transport receptor‐independent pathways exist as it was described for, for example, IκBα (Sachdev et al., 2000).

The import is initiated by binding of importin to its corresponding nuclear import motif. Via multiple interactions, these complexes pass the hydrophobic mesh in the central pore channel (Yoshimura et al., 2014). Upon interacting with Nup153, dissociation of the cargo from the importin occurs through binding of the Ras‐like small GTPase Ran in its GTP‐bound form (Walther et al., 2001).

Nuclear export follows a similar principle in which a nuclear export signal (NES) containing cargo traverses the NPC toward the cytoplasm. NESs are characterized by a hydrophobic profile, as is found on the HIV Rev protein (LPPLERLT) (Fischer et al., 1995). NESs allow binding of exportins, such as Chromosomal Maintenance 1 (CRM1) in complex with RanGTP (Kehlenbach et al., 1999; Petosa et al., 2004). After translocation through the NPC, export complexes reach cytoplasmic filaments where Nup214 binds to CRM1 allowing the closely localized Nup358‐bound RanGAP to trigger the GTPase function of Ran, catalyzing the hydrolysis of RanGTP to RanGDP. This results in a conformational change and the dissociation of the transport‐cargo complex (Hutten et al., 2008; Mahadevan et al., 2013; Ritterhoff et al., 2016; Wälde et al., 2012).

### 2.3 Nuclear entry of parvoviruses through the NPC

Although the molecular details of parvoviral nuclear import remain controversial, it has been suggested that intact capsids enter the nucleus followed by genome release at some distance from the NE (Bernaud et al., 2018; Mäntylä et al., 2018). Previous studies on MVM demonstrated that the nuclear release of parvoviral genomes occurs without complete disassembly of the capsids (Cotmore et al., 1999; Ros et al., 2006; Ros & Kempf, 2004). However, fast diffusion of intranuclear CPV capsid fragments demonstrates the presence of disassembled capsids (Mäntylä et al., 2018). Irrespectively to the intranuclear fate of capsids, which is linked to the unknown mechanism of genome release, they may have to be primed for genome release prior to nuclear import as B19V capsid uncoating is enhanced by cytoplasmic depletion of divalent cations (Caliaro et al., 2019).

Single particle imaging demonstrated that the first nuclear AAV‐2 capsids are detected already 15 min after adding the viral particles to cell culture (Seisenberger et al., 2001) although others reported that more than 2 hours are needed for nuclear capsid arrival (Bartlett et al., 2000; Sonntag et al., 2006; Zhong et al., 2008). The entry of intact capsids was also observed for CPV either after infection or after cytoplasmic microinjection of viral particles; the latter observation indicating that acidification and subsequent structural changes are not essential for nuclear entry (Harbison et al., 2009; Suikkanen, Antila, et al., 2003b; Vihinen‐Ranta et al., 2002). However, these microinjections were performed using parvovirus‐susceptible cells and the technically caused leakage of capsids from the needle prior to injection leads to exposure of capsids to the cell exterior thus initiating parallel infections.

**FIGURE 1:**
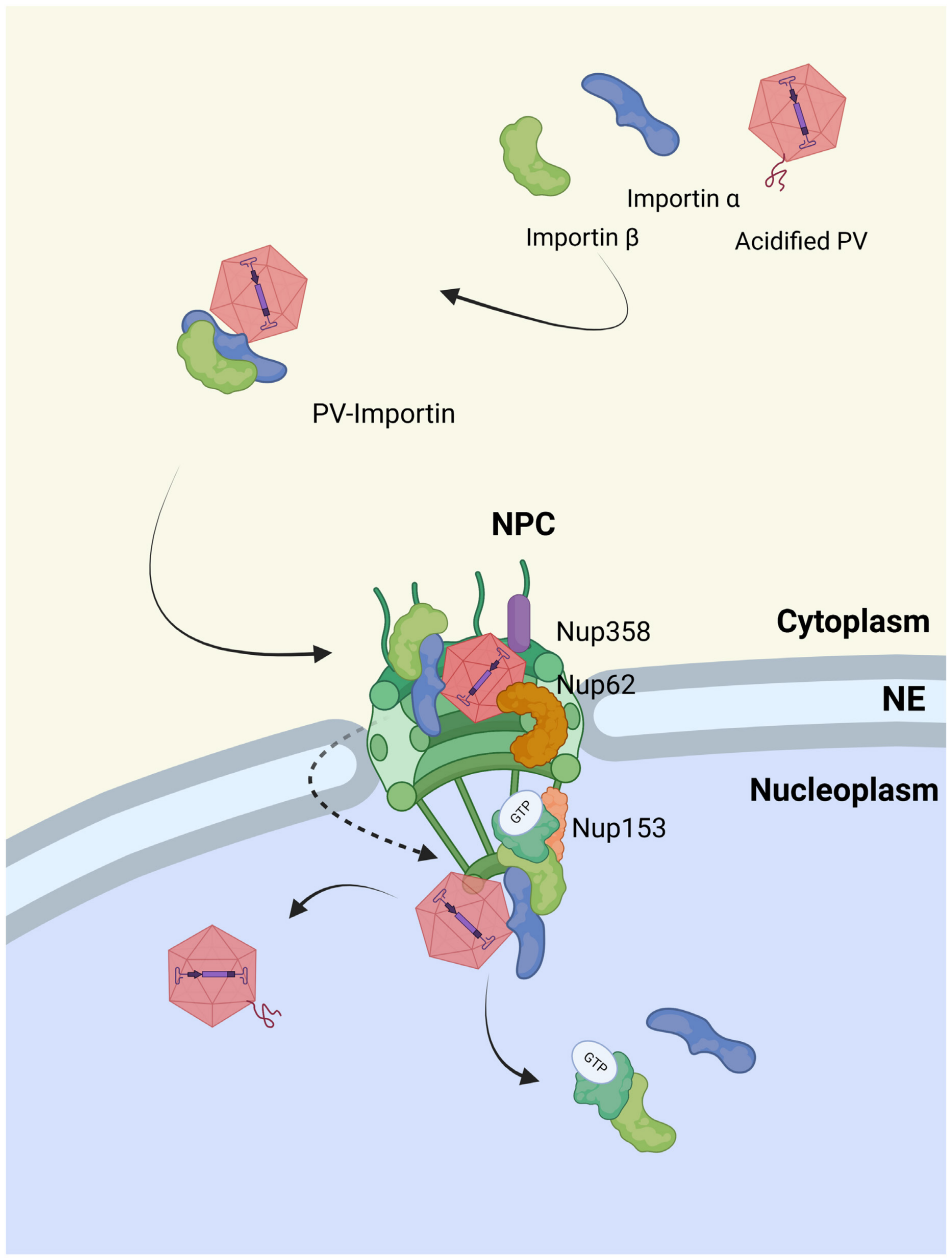
Nuclear entry of parvoviruses through the NPC. Cytoplasmic parvoviruses (PVs) that have undergone structural changes within the endosome bind to importin α (KPNA) /importin β (KPNB). This allows transport through cellular nuclear pore complexes (NPCs). Upon reaching the nuclear basket, the PV‐importin complex dissociates, releasing the capsid into the nucleoplasm. Figure created with BioRender.

**FIGURE 2:**
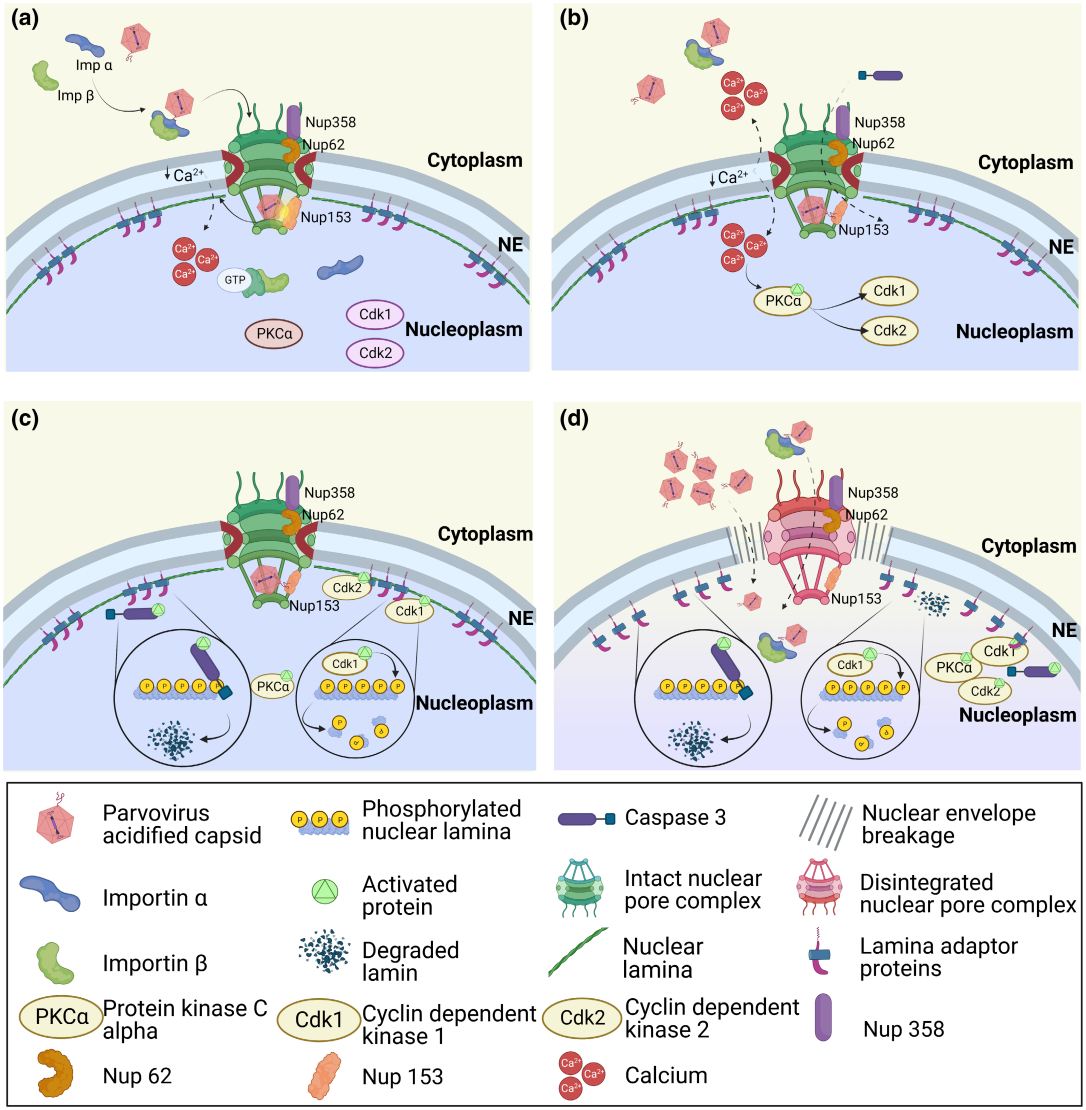
Entry through the NE by increased nuclear envelope permeability. (a) Parvovirus capsids bound to importins (KPNA: Importin α/KPNB: Importin β) bind to Nups. The binding triggers exposure of PLA_2_ on VP1u inducing calcium efflux. (b) the release of calcium activates PKCα, which activates cdk2/cdk1. Caspase 3 is also activated. (c) Hyper‐phosphorylation of Lamin B by kinases as well as Lamin B‐cleavage by caspase 3 leads to its local degradation. (d) The formation of transient holes allows entry of NPC‐bound or cytosolic capsids or capsid‐importin complexes. Figures created with BioRender.

Two possible pathways of how parvoviral capsids enter the nucleus have been proposed: a “classical” entry passing the NPCs using the NLS on VP1u, which binds to nuclear import factors of the importin family (Table 1). This would allow the capsids to pass the NPC due to their small diameter which is below the 40 nm size limit of the NPC (Panté & Kann, 2002) (Figure 1). Alternatively, parvoviral capsids may enter the nucleus through transient holes in the NE, which are induced by their interaction with Nups (Porwal et al., 2013) (Figure 2). Due to the low efficiency of all parvoviruses, it remains not fully evident which pathway leads to progeny infection, and a combination of both pathways appears possible.

**TABLE 1:** Key facts on nuclear entry of parvovirus capsids

	Nuclear entry requirements	Active transport NPC	Interaction with Nups	NEBD
Autonomous parvovirus	**B19V:** Potential depletion of capsid‐associated divalent cations for uncoating (Caliaro et al., 2019) **CPV** and **H‐1PV**: Ca^2+^ release for NE disruption (Porwal et al., 2013)	**CPV**: Capsids recruit importin β to form capsid‐importin β complex (Mäntylä et al., 2020) capsid‐importin β complex is transported into the nucleus (Mäntylä et al., 2018)	**H‐1PV**: Coprecipitation with Nup358, Nup153 and Nup62 (Porwal et al., 2013) Interaction with Nups may trigger PLA_2_ exposure to induce initial Ca^2+^ release for NE disintegration (Porwal et al., 2013)	**MVM:** NE invagination and redistribution of lamin A/C (Cohen et al., 2006) **H‐1PV:** Ca^2+^ release triggers activation of mitotic factors (PKC, cdk2/cdk1 and caspase 3) for NE disintegration by local lamin B depolymerization. No soluble cytosolic factors needed in permeabilized cells (Porwal et al., 2013) **CPV** failed to infect cells preloaded with hepatitis B capsids by microinjection (Porwal et al., 2013)
Dependo‐parvovirus	**AAV2:** Capsid acidification (pH 5.2) and Ca^2+^ release was required for NE disruption (Porwal et al., 2013)	**AAV2:** Three NLS‐like motifs in VP1 and VP2 essential for infection (Johnson et al., 2010). Three PDZ‐motifs on VP1u essential for nuclear entry and infection (Popa‐Wagner et al., 2012). Labeled capsids pass‐through NPC, no evidence of NE disintegration: (Kelich et al., 2015) **rAAV2:** Interaction with importin β with or without interaction with importin α (Nicolson & Samulski, 2014)	**AAV2** coprecipitates with Nup358, Nup153 and Nup62 (Porwal et al., 2013)	NEBD limited to microinjected **AAV2** capsid exposed to pH 5.2 (Porwal et al., 2013) NE invagination showed by EM (Cohen et al., 2006; Cohen & Panté, 2005)

As mentioned before, acidification leads to exposure of VP1u, which comprises a NLS with basic residues as shown for CPV (Cotmore et al., 1999, 2010; Vihinen‐Ranta et al., 2002). Similarly, the externalized N‐terminus of AAV2 VP1 and VP2 proteins comprise three NLS‐like motifs which are essential for the progression of infection (Grieger et al., 2006; Hoque et al., 1999; Johnson et al., 2010; Sonntag et al., 2006), as well as three PDZ‐motifs crucial for nuclear entry and infection (Popa‐Wagner et al., 2012). These NLSs may not only contribute to the nuclear transport of the capsids but also to the transport of capsid proteins, required for nuclear assembly of progeny parvoviruses. In fact, VP1/VP2 trimers of MVM are actively imported although using importin β ‐independent pathway (Riolobos et al., 2006). Of note, the nuclear import of these trimers requires VP1/VP2 phosphorylation by Raf‐1 kinase during entry of the cell into S phase, which may contribute to MVM specificity for transformed cells (Lombardo et al., 2000; Riolobos et al., 2006).

More direct evidence for importin β‐recruitment on assembled capsids was found in time‐lapse microscopy of CPV after infection, showing that the formation of importin β‐CPV‐complexes slows the diffusion of cytoplasmic CPV capsids (Mäntylä et al., 2018, 2020). Further, importin β‐CPV capsid complexes are transported simultaneously through the NE (Mäntylä et al., 2018), which is consistent with data on rAAV‐2 nuclear import, which depends on interaction with importin β alone or in complex with importin α (Nicolson & Samulski, 2014). However, at least some nuclear capsids remain decorated with importin β (Mäntylä et al., 2018) arguing against a classical nuclear import of at least a fraction of capsids, as importin β becomes removed from the cargo within the nuclear basket during classical NLS‐dependent nuclear import.

However, the number of detected intranuclear capsids is very low but in agreement with the small number of nuclear replication compartment foci detected in early stages of MVM and CPV infection (Ihalainen et al., 2007; Ruiz et al., 2006). It can be thus not excluded that only a minor fraction of parvoviral capsids initiates infection.

### 2.4 Entry through the NE by increased nuclear envelope permeability

Various parvoviruses exhibit a unique feature in that they permeabilize transiently the NE, as it was shown for H‐1PV, CPV, and AAV2 (Cohen et al., 2006; Cohen & Panté, 2005; Popa‐Wagner et al., 2012; Porwal et al., 2013) (Table 1). This nuclear envelope break‐down (NEBD) occurs within minutes after capsid exposure to nuclei, being in agreement with a rapid passage of the capsid into the nucleus observed in infection. This led to the hypothesis that these holes in the nuclear envelope allow nuclear entry of intact capsids (Figure 2). Mechanistically, parvoviral NEBD shows similarities to mitosis in that Ca^++^, released from the lumen between inner and outer nuclear membrane, initiates activation of PKCα, which activates Cdk2 and/or Cdk1, followed by activation of caspase 3 (Figure 2b). The activation of the kinases allows the hyper‐phosphorylation of lamin B, which was described to cause local lamin (Cohen et al., 2006). Such depolymerization is required for open holes of up to 190 nm (Porwal et al., 2013), which are large enough to allow entry of the capsids or even larger complexes as capsid‐importin complexes. The role of caspase 3 is in the proteolytic cleavage of lamin B and not in the direct disruption of the nuclear membranes (Cohen et al., 2006, 2011; Cohen & Panté, 2005) (Figure 2c). Caspase 3 is upregulated and activated just prior to mitosis (Hsu et al., 2006), being in concordance with its function during parvoviral‐mediated NEBD.

Pore formation depends on interaction with the NPC in particular by capsid binding to at least three Nups (Nup358, Nup153, and Nup62). Blocking AAV2 or H‐1PV interaction with Nup153 by hepatitis B virus capsids, which specifically interacts with Nup153 (Schmitz et al., 2010) inhibited NEBD. The relevance of this finding for infection was later confirmed by CPV, which failed to infect cells preloaded with hepatitis B virus capsids by microinjection (Mäntylä et al., 2020). As Nup153 is localized in the nuclear basket close to the inner ring of the NPC, these observations indicate that the parvoviruses should be associated with importins in order to reach the nuclear side of the NPC (Figure 2a). Furthermore, NEBD was accelerated when the capsids were pre‐acidified and neutralized implying the need of VP1u exposure. In fact, PLA_2_ exposure could also be achieved by direct interaction of parvoviral capsids (AAV2 and H‐1PV) with Nups leading to the hypothesis that the accessible PLA_2_ domain triggers the initial Ca^++^ efflux. However, PLA_2_ activity on MVM capsids has not been reported to be involved in causing NE disruption (Cohen et al., 2011) and other mechanisms causing permeabilization cannot be excluded. This includes amphipathic helices identified on VP1u (Leisi et al., 2016) as they permeabilize membranes, which was as shown for endosomal escape of adenoviruses (Wiethoff & Nemerow, 2015), or the PDZ domains, which exhibit membrane affinity (Fanning & Anderson, 1999) and induce membrane curvature (Herlo et al., 2018).

In summary, there are two seemingly contradictory models of nuclear import of parvoviral capsids but both rely on interaction with the NPC, which was also demonstrated by single particle tracking of rAAV (Junod et al., 2021; Kelich et al., 2015). Numerous data support that this interaction is mediated by importin β, however, the interaction between importin α and the NLS exposed on VP1u on capsid surface is not well understood. Nuclear import of microinjected capsids (Harbison et al., 2009; Suikkanen, Aaltonen, et al., 2003a) suggests that a sub‐fraction of capsids might expose their VP1us without acidification. Further, it cannot be totally excluded that the nuclear capsids after microinjection are derived from infection occurring in parallel.

Differences between the models comprise later events once the capsids arrive on the nuclear side of the NPC. While the classical import model favors dissociation of the importins from the capsids and diffusion of the latter deeper into the nucleus, the NEBD model supports interaction with Nup153 possibly after importin β dissociation, disintegration of the NE and entry of the capsid. These could be either cytosolic capsids (eventually importin β‐bound) or the capsids that have initiated the NEBD, likely after NPC‐dissociation which is mediated by cdk‐1 (Kutay et al., 2021). However, as long as it remains unknown which capsids initiate infection, none of the models can be excluded.

## 3 NUCLEAR EGRESS

Once parvovirus genome has entered the nucleus, successful replication depends on cell entry into the S phase. The S phase‐dependent activation of DNA replication machinery is needed to provide the resources necessary for viral replication. These cellular factors include DNA polymerase δ required for conversion of ssDNA to dsDNA template for viral gene transcription (Cotmore & Tattersall, 2013). The progression to the S phase is accompanied by virus‐induced cellular DNA damage, ataxia telangiectasia mutated (ATM)‐dependent DNA damage response (DDR) and pre‐mitotic cell cycle arrest in MVM infection (Adeyemi et al., 2010; Cotmore & Tattersall, 2013; Ruiz et al., 2011). In AAV infection, cytotoxic viral Rep proteins induce S‐phase arrest (Berthet et al., 2005; Saudan et al., 2000), and UV‐treated AAV particles evoke ATM‐ and Rad3‐related kinase (ATR)‐dependent DDR characterized by accumulation of cells in the late S and/or G2 phases (Jurvansuu et al., 2005; Raj et al., 2001; Schwartz et al., 2009; Winocour et al., 1988). Preventing cell entry from G2 phase to mitosis maintains nuclear structure thereby allowing the prolonged assembly of new virions (Adeyemi & Pintel, 2014; Chen et al., 2010; Morita et al., 2003). The empty capsids are formed in the nucleus, and they mature into DNA‐filled capsids at the late S/G2 phase (Gil‐Ranedo et al., 2015). After AAV capsid assembly, involving capsid accumulation in nucleoli (Sonntag et al., 2010; Wistuba et al., 1997), targeting of viral ssDNA to viral capsid is mediated by Rep proteins (Bleker et al., 2006; Dubielzig et al., 1999).

Viral infection elicits various responses in the host cell which can lead to plasma membrane ruptures, formation of membrane vesicles, nuclear fragmentation, and finally to cell lysis (Labbé & Saleh, 2008). The cellular egress of many non‐enveloped viruses is a passive process which relies on cell lysis to release viral progeny into the extracellular space (Daeffler et al., 2003; Georgi & Greber, 2020; Tollefson et al., 1996). The major form of cell death described for parvoviruses is apoptosis, however, also necrosis has been detected (Chen & Qiu, 2010; Nykky et al., 2010).

In apoptotic cells, the NE permeability is regulated by caspase‐dependent and ‐independent alterations of NPCs and caspase‐dependent cleavage of lamins and other NE proteins (Ferrando‐May, 2005; Kihlmark et al., 2004; Roehrig et al., 2003; Strasser et al., 2012). As described earlier, nuclear entry of parvovirus capsids is accompanied by the NE disintegration and activation of the key enzymes of mitosis (Porwal et al., 2013). However, nuclear microinjection of H‐1PV capsids does not induce NEBD making it unlikely that this entry‐related mechanism is required for capsid egress from the nucleus.

During parvovirus infection, the disintegration of host DNA is followed by DNA damage response and activation of apoptosis (Adeyemi et al., 2010; Chen & Qiu, 2010). The cell death events are mediated by apoptotic caspases (Roos & Kaina, 2006). CPV‐infected cells have a relatively long lifespan even though the initiator caspases 8 and 9, and effector caspases 3 and 7 are activated early in infection and remain active until very late in infection, until 48–72 hpi (Nykky et al., 2010). Analysis of infected cells has indicated that capsids are released from host cells already at 12 hpi (Zhao et al., 2016). These observations support the model that viral capsids egress the nucleus and the host cell prior to apoptosis‐induced cell lysis. After nuclear exit cytoplasmic MVM progeny capsids are transported through COPII‐vesicles of ER and cisternae of Golgi and continue toward the cellular periphery in lysosomal/late endosomal vesicles. The vesicular capsid transport and cellular exocytosis depend on gelsolin‐induced degradation of actin (Bär et al., 2008).

**FIGURE 3:**
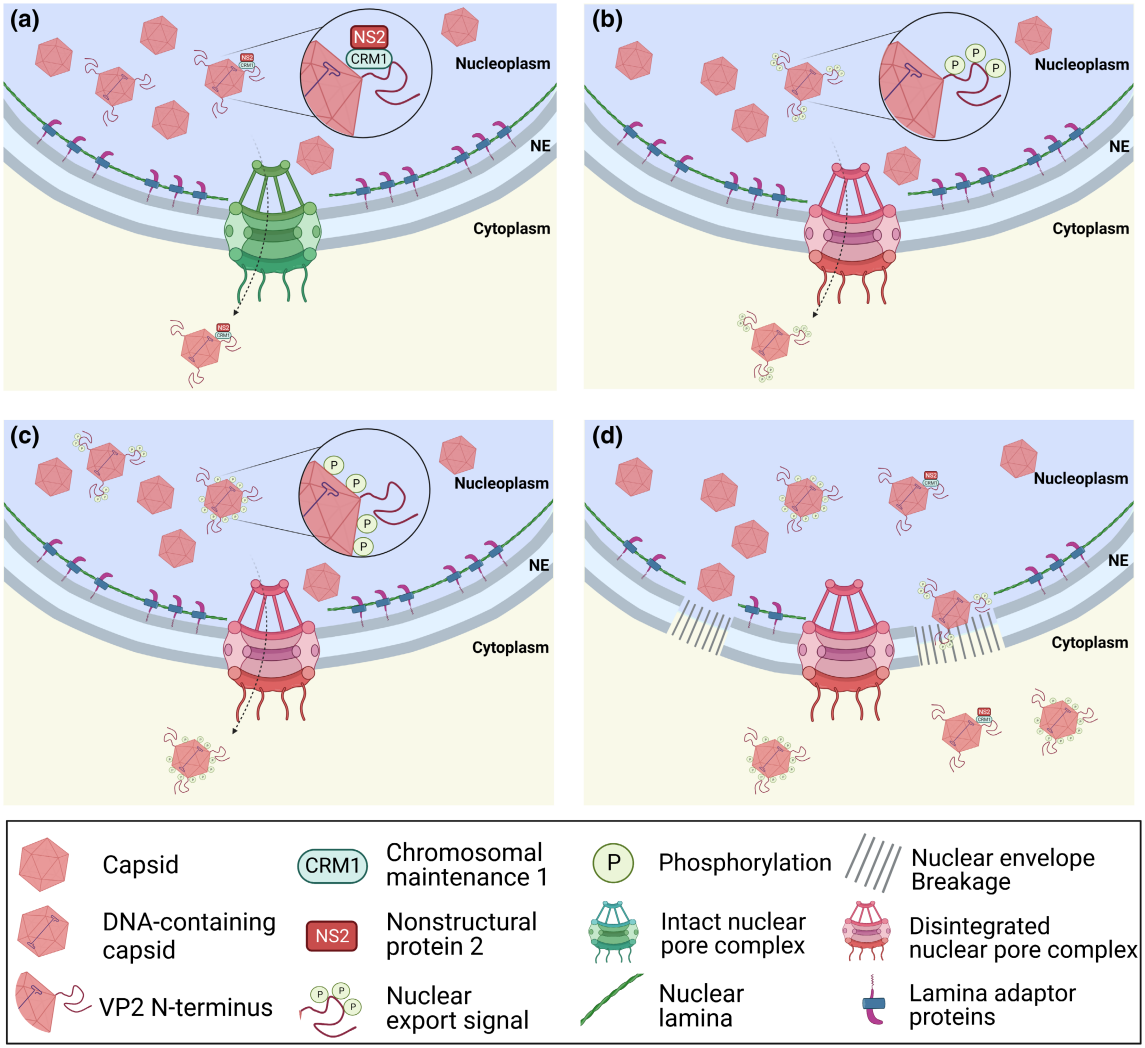
Nuclear egress of progeny capsids. Packaging of viral genomes inside capsids causes a conformational change exposing the VP2 N‐terminal on the capsid surface. (a) MVM capsids are actively exported out of the nucleus through NPCs mediated by the interaction between NS2 NES with CRM1 (Bodendorf et al., 1999; Eichwald et al., 2002; Engelsma et al., 2008; Fornerod et al., 1997; Maroto et al., 2004; Miller & Pintel, 2002). (b) The phosphorylation of the exposed VP2 N‐terminal end on the capsid surface acts as a nuclear export signal enhancing capsid export out of the nucleus (Maroto et al., 2004). (c) Phosphorylation of the capsid surface enhances capsid export (Wolfisberg et al., 2016). (d) Activation of apoptosis and necrosis affect the structure of the nuclear lamina, and capsids are released to the cytoplasm in late infection (Chen & Qiu, 2010; Nykky et al., 2010, (Wolfisberg et al., 2016). Figures created with BioRender.

In contrast to these cell‐destruction‐based exit mechanisms, which were previously thought to be the main pathway for progeny parvoviral egress, more recent evidence supports that parvoviruses are also able to actively egress the nucleus into cytosol before passive release through cell lysis at the final stage of the infection occurs (Table 2). Active translocation has been previously shown for MVM, which utilizes the CRM1‐mediated active nuclear export pathway for nuclear exit of capsids through the NPC (Eichwald et al., 2002; Engelsma et al., 2008) (Figure 3a). CRM1, also called exportin 1, is a versatile nuclear export receptor which shuttles between the nucleus and cytoplasm (Fornerod et al., 1997) and translocates multiple cargoes including ribosomal subunits (Ho et al., 2000; Moy & Silver, 1999; Thomas & Kutay, 2003). The binding of CRM1 to the cargo is promoted by RanGTP (Koyama & Matsuura, 2010) and mediated by NES. For nuclear export of MVM capsids, CRM1 interacts with the NES in NS2 in a RanGTP‐independent manner (Bodendorf et al., 1999; Eichwald et al., 2002; Engelsma et al., 2008; Fornerod et al., 1997; Maroto et al., 2004; Miller & Pintel, 2002).

**TABLE 2:** Key facts on nuclear egress of parvovirus capsids

	NS2‐CRM1 interaction	Active transport NPC	Phosphorylation	Apoptosis
Autonomous parvoviruses	**MVM**: CRM1 interacts with the NES in NS2 (Bodendorf et al., 1999; Eichwald et al., 2002; Engelsma et al., 2008; Fornerod et al., 1997; Maroto et al., 2004; Miller & Pintel, 2002)	**MVM:** NS2 NES is required for active nuclear export of progeny viruses (Engelsma et al., 2008)	**MVM**: Phosphorylation of serine residues in the exposed N ‐terminal end of VP2 functions as a NES contributing to active export (Maroto et al., 2004). Phosphorylation of the capsid surface enhances nuclear export capacity. VP2 N‐terminal phosphorylation is involved in passive release, but not required for active transport (Wolfisberg et al., 2016)	**CPV**: Infection activates caspases 9,8, 3/7. (Nykky et al., 2010) **Minute virus of canines (MVC):** Infection‐induced DNA damage leads to p53‐dependent cell death (Chen & Qiu, 2010)
Dependo‐parvovirus				**AAV:** Activities of helper virus leads to cell lysis and viral exit (Meier et al., 2020, Smith & Enquist, 2002)

Several findings, however, support CRM1‐independent nuclear capsid export, being thus most likely NS2‐independent. Similar to nuclear import of MVM VP1/VP2 trimers, nuclear egress of MVM capsids is enhanced by Raf‐1 kinase‐mediated phosphorylation of three serine residues in the N‐terminus of VP2 on capsid surface (Maroto et al., 2004) (Figure 3b). This pathway relies on exposure of the N‐terminal domain of VP2, which is exposed in DNA‐containing parvovirus capsids during their maturation (Agbandje‐McKenna et al., 1998; Kaufmann et al., 2008; Kontou et al., 2005; Sánchez‐Martínez et al., 2012; Tsao et al., 1991). Moreover, the phosphorylation of the capsid surface residues has been linked to nuclear export capacity prior to the passive release by cell lysis. Although conformational change of the VP2 N‐ terminus on the capsid surface was required for phosphorylation, the VP2 N‐terminus was dispensable for nuclear capsid egress (Wolfisberg et al., 2016) (Figure 3c.) Non‐phosphorylated capsids exit the nucleus passively upon NE damage during apoptosis (Figure 3d). The cellular and nuclear egress of AAV was earlier thought to rely on cell lysis caused by overexpression of helper virus, adenovirus or herpesvirus, proteins (Meier et al., 2020; Smith & Enquist, 2002). Recently, the presence of viral membrane‐associated accessory protein (MAAP) was observed for AAV at the late stages of infection. MAAP is located in the plasma membrane and in the nuclear periphery (Galibert et al., 2021; Ogden et al., 2019). This protein is a viral egress factor, which also promotes AAV capsid association with extracellular vesicles (Elmore et al., 2021).

## 4 CONCLUDING REMARKS

The versatile therapeutic potential of parvovirus has researchers focused on understanding the full mechanism of infection. In gene therapy, for which an efficient delivery of modified parvoviral vectors (mostly AAV) is crucial, nuclear entry seems to be a bottleneck and detailed knowledge may help improving their clinical administration. Similar to nuclear entry, the studies of viral egress have shown also controversial results. Improving the knowledge on export may assist oncolytic therapy using autonomous parvoviruses, as their potential depends upon efficient spread.

## ETHICS STATEMENT

The work presented here did not include human or animal subjects nor human or animal material or data. Thus, no formal consent or approval was necessary.

## Data Availability

Data sharing not applicable ‐ no new data generated.
